# Global analysis of human duplicated genes reveals the relative importance of whole-genome duplicates originated in the early vertebrate evolution

**DOI:** 10.1186/s12864-016-2392-0

**Published:** 2016-01-22

**Authors:** Debarun Acharya, Tapash C. Ghosh

**Affiliations:** Bioinformatics Centre, Bose Institute, P 1/12, C.I.T. Scheme VII M, Kolkata, 700054 West Bengal India

**Keywords:** Small-scale duplication, Whole-genome duplication, Functional divergence, Gene essentiality, Disease genes, Protein multifunctionality, Evolutionary rate

## Abstract

**Background:**

Gene duplication is a genetic mutation that creates functionally redundant gene copies that are initially relieved from selective pressures and may adapt themselves to new functions with time. The levels of gene duplication may vary from small-scale duplication (SSD) to whole genome duplication (WGD). Studies with yeast revealed ample differences between these duplicates: Yeast WGD pairs were functionally more similar, less divergent in subcellular localization and contained a lesser proportion of essential genes. In this study, we explored the differences in evolutionary genomic properties of human SSD and WGD genes, with the identifiable human duplicates coming from the two rounds of whole genome duplication occurred early in vertebrate evolution.

**Results:**

We observed that these two groups of duplicates were also dissimilar in terms of their evolutionary and genomic properties. But interestingly, this is not like the same observed in yeast. The human WGDs were found to be functionally less similar, diverge more in subcellular level and contain a higher proportion of essential genes than the SSDs, all of which are opposite from yeast. Additionally, we explored that human WGDs were more divergent in their gene expression profile, have higher multifunctionality and are more often associated with disease, and are evolutionarily more conserved than human SSDs.

**Conclusions:**

Our study suggests that human WGD duplicates are more divergent and entails the adaptation of WGDs to novel and important functions that consequently lead to their evolutionary conservation in the course of evolution.

**Electronic supplementary material:**

The online version of this article (doi:10.1186/s12864-016-2392-0) contains supplementary material, which is available to authorized users.

## Background

Gene duplication is a key source for generating new gene copies from pre-existing ones [1–3]. These newly-made gene copies are initially functionally redundant and relieved from selective pressure, and may adapt themselves to new functions [2, 4–6]. Thus, many of the previous studies concluded gene duplication as the primary guiding force of organism evolution for providing raw genetic materials for genome evolution [1, 2, 7]. Although, the retention of duplicated genes is not a trouble-free process and most of the duplicates become nonfunctionalized and/or lost from the genome [2], whereas others become fixed within the genome in course of evolution. The retention of duplicates might be initially favourable due to circumstances like increased gene dosage advantage, where the duplication and subsequent increase in the gene product may be advantageous to the organism [5, 8]. Additionally, gene duplicates may serve as backup copies capable of functional compensation upon gene deletion [9] and provide increased genetic robustness against deleterious mutations [10], but their maintenance requires stringent regulation in gene dosage [11, 12] or expression patterns [13–16]. That apart, the duplicates may either diverge at the subcellular protein localization [17] or share the ancestral function [18] after complementary degenerative mutations (subfunctionalization) [19] or adapt to new functions (neofunctionalization) [2]. Furthermore, there are also subtle differences in the extent of gene duplication. In most of the cases, duplication involves a single gene and termed as small-scale duplication (SSD), whereas, large-scale duplications may involve many genes, chromosomal segments or even the entire genome, with the latter being known as whole-genome duplication (WGD) [20]. Although small-scale duplication can occur at any time and may be retained in course of evolution, there are a few evidences of whole genome duplication in eukaryotic organisms, being most common and widely studied in the evolution of plant genome [21–24]. Many previous studies highlighted the evidence of an ancient WGD in the yeast genome [25–27]. Additionally, evidence of two rounds of whole-genome duplication was also prominent in the early vertebrate evolution [28–33], which provides the raw materials for increasing genome and organism complexity and extensive species diversity [29, 31] and hence, is an important process in vertebrate evolution [30, 31].

However, as genes’ functions are mainly mediated by their encoded proteins, which primarily function with the association of other such proteins [34], the proper functioning of a gene depends on the stoichiometric balance of the proteins participants. The retention of duplicated genes creates a stoichiometric disparity in the protein-protein interaction network, with the duplicated genes producing more proteins than the non-duplicated ones [35–37]. The two extent of duplication affect their associated protein-interaction network differentially [20, 38–41]. In WGD, the whole PPI network becomes simultaneously duplicated, and the stoichiometric balance of the participant proteins remains the same; whereas in SSD, the duplicated gene tends to form more protein in contrast to the non-duplicated interacting partners, thereby creating an imbalance in the whole PPI network. Therefore, in general, whole-genome duplicates are expected to be retained intact within the genome [39].

Most of the studies highlighting gene duplication compared the attributes of duplicated genes with that of singletons [10, 42, 43]. This raised an important question − are all duplicates equal in their genomic and evolutionary characteristics? With the well-established gene duplication data in yeast, it became possible to identify the duplicates originated from whole-genome duplication as well as those from small-scale duplication [25]. Comparing these two distinct duplicate groups, researchers observed quantifiable differences in yeast [20, 41, 44]. They found that the yeast WGDs are functionally more similar than SSD genes, which is independent of their sequence similarity [20, 44]. Additionally, yeast SSDs also diverge more at their subcellular localization than the WGDs [41]. Also, yeast SSD genes were found to contain a higher proportion of essential genes than WGD genes [20, 44].

The occurrence of two rounds of whole-genome duplication in early vertebrate lineage [28–33] and the subsequent detection of traces of these whole-genome duplicates in human [32, 39, 45] lead us to differentiate the genomic and evolutionary attributes of human small-scale and whole-genome duplicates. As the human WGDs stem from the ancient two rounds of genome duplication that had occurred in early vertebrates, it can be stated that these human duplicates became subjected to more evolutionary pressure due to their long term evolutionary exposure than that in yeast. Therefore, our study will explore the relative importance and the long-term fate of these whole-genome duplicates that had originated during the early vertebrate evolution in contrast to the duplicates originating spontaneously at small-scale.

## Results

### Functional similarity of human SSD and WGD genes

**Fig. 1:**
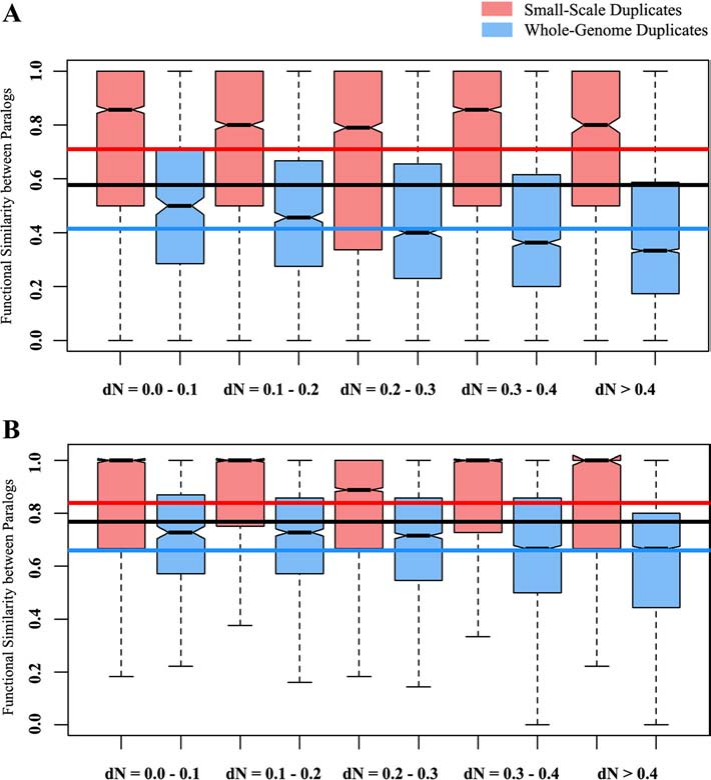
Functional similarity between human small-scale and whole-genome duplicate pairs. The SSDs are represented in brick red and WGDs are represented in blue. The red and blue lines represent the mean functional similarity of SSD and WGD pairs, respectively. The black line represents the mean functional similarity of all human duplicates. The functional similarities between different dN ranges were calculated using both GO domains. **a**. Biological Process and **b**. Molecular Function (For every dN range, *P* < 0.05). For exact P-values, refer Table 1

The functional similarities between each pair of human small-scale and whole-genome duplicates were calculated using the Gene Ontology (GO) annotation from the biomart interface of Ensembl (version 77) [46], using GO domains ‘biological process’ as well as ‘molecular function’. We obtained a higher functional similarity in small-scale duplicates than the whole-genome duplicated group (Table 1). However, the functional diversification of paralogs is dependent on their nonsynonymous nucleotide substitution per nonsynonymous site (dN), and the whole-genome duplicates tend to have a higher dN value the small-scale duplicates, for being evolutionarily more ancient. Therefore, we binned our dataset according to different dN ranges (nonsynonymous nucleotide substitution per nonsynonymous site) (see Materials and methods) and compared the functional similarity between SSD and WGD duplicate pairs. This approach is similar to that adopted by Hakes et al. [20]. We found that SSD duplicate pairs are functionally more similar than the WGD pairs in each dN range (Table 1) considering both their involvement in biological processes and molecular function (Fig. 1). In other terms, human WGD pairs were found to be functionally more divergent, independent of their sequence divergence.

**Table 1:** Differences between the properties of human small-scale and whole-genome duplicate pairs in different dN ranges. Pair wise two-tailed *Mann–Whitney U test* were used to compare the means of SSD and WGD pairs within each group

Parameter Measured	Database used	Overall			dN 0.0–0.1			dN 0.1–0.2			dN 0.2–0.3			dN 0.3–0.4			dN > 0.4		
		SSD	WGD	P-value	SSD	WGD	P-value	SSD	WGD	P-value	SSD	WGD	P-value	SSD	WGD	P-value	SSD	WGD	P-value
Functional Similarity between paralogs	Shared GO Terms for Biological Process	x̄= 0.710	x̄= 0.415	<1.00 × 10^−6^	x̄= 0.734	x̄= 0.499	2.325 × 10^−52^	x̄= 0.720	x̄= 0.476	5.925 × 10^−123^	x̄= 0.657	x̄= 0.440	2.892 × 10^−120^	x̄= 0.726	x̄= 0.413	1.397 × 10^−274^	x̄= 0.710	x̄= 0.391	1.983 × 10^−135^
		*N* = 14742	*N* = 12022		*N* = 3640	*N* = 414		*N* = 2754	*N* = 1140		*N* = 3328	*N* = 2002		*N* = 4264	*N* = 2192		*N* = 756	*N* = 6274	
	Shared GO Terms for Molecular Function	x̄= 0.840	x̄= 0.659	<1.00 × 10^−6^	x̄= 0.850	x̄= 0.724	6.077 × 10^−47^	x̄= 0.856	x̄= 0.706	1.075 × 10^−129^	x̄= 0.810	x̄= 0.696	1.814 × 10^−96^	x̄= 0.846	x̄= 0.677	4.976 × 10^−218^	x̄= 0.826	x̄= 0.628	5.072 × 10^−107^
		*N* = 18584	*N* = 12392		*N* = 4668	*N* = 410		*N* = 3510	*N* = 1188		*N* = 4300	*N* = 2076		*N* = 5246	*N* = 2250		*N* = 860	*N* = 6468	
Shared Subcellular Compartment of paralogs	GO Cellular Component	x̄= 0.782	x̄= 0.541	<1.00 × 10^−6^	x̄= 0.816	x̄= 0.579	5.341 × 10^−76^	x̄= 0.788	x̄= 0.581	5.652 × 10^−119^	x̄= 0.740	x̄= 0.541	3.344 × 10^−139^	x̄= 0.781	x̄= 0.555	1.421 × 10^−215^	x̄= 0.777	x̄= 0.527	1.156 × 10^−100^
		*N* = 15248	*N* = 12198		*N* = 3790	*N* = 380		*N* = 2914	*N* = 1162		*N* = 3444	*N* = 2036		*N* = 4356	*N* = 2228		*N* = 744	*N* = 6392	
Gene expression profile similarity between paralogs	Human Protein Atlas	x̄= 0.403	x̄= 0.193	<1.00 × 10^−6^	x̄= 0.615	x̄= 0.254	1.558 × 10^−63^	x̄= 0.414	x̄= 0.253	1.774 × 10^−42^	x̄= 0.307	x̄= 0.191	7.331 × 10^−32^	x̄= 0.316	x̄= 0.190	1.131 × 10^−34^	x̄= 0.322	x̄= 0.179	1.308 × 10^−17^
		*N* = 11726	*N* = 13060		*N* = 2588	*N* = 426		*N* = 2758	*N* = 1226		*N* = 2834	*N* = 2158		*N* = 3042	*N* = 2366		*N* = 504	*N* = 6884	
	Expression Atlas	x̄= 0.450	x̄= 0.216	<1.00 × 10^−6^	x̄= 0.508	x̄= 0.284	1.032 × 10^−30^	x̄= 0.457	x̄= 0.280	5.953 × 10^−53^	x̄= 0.430	x̄= 0.216	1.377 × 10^−105^	x̄= 0.420	x̄= 0.219	5.471 × 10^−96^	x̄= 0.394	x̄= 0.199	5.735 × 10^−36^
		*N* = 15404	*N* = 13072		*N* = 3628	*N* = 422		*N* = 3458	*N* = 1220		*N* = 3792	*N* = 2166		*N* = 3922	*N* = 2370		*N* = 604	*N* = 6894

### Subcellular localization of SSD and WGD pairs

**Fig. 2:**
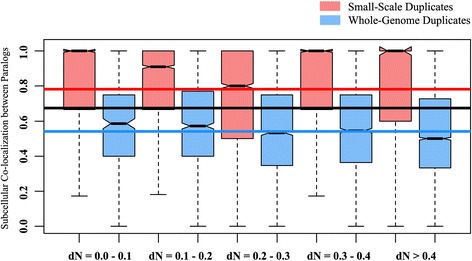
Subcellular co-localization between human small-scale and whole-genome duplicate pairs. The SSDs are represented in brick red and WGDs are represented in blue. The red and blue lines represent the mean functional similarity of SSD and WGD pairs, respectively. The black line represents the mean functional similarity of all human duplicates (For every dN range, *P* < 0.05). For exact P-values, see Table 1

In addition to the functional divergence, insight into the function of a gene is associated with the location of its encoded protein within the cell at the subcellular level. Many previous studies reported that gene duplication and the functional redundancy of duplicates can often be neutralized at the protein level by the subcellular protein compartmentalization [17, 47, 48]. Therefore, we also considered the subcellular localization of their encoded proteins as an alternative and/or associated mechanism beside functional divergence of the duplicated genes. The localization of the protein can be obtained by using the Gene Ontology (GO) terms under the GO domain ‘Cellular Component’ against its gene identifier. The shared cellular component between the paralogous copies of all SSD and WGD genes were calculated (see Materials and methods). We observed an overall higher subcellular compartment sharing of SSD pairs than that of WGD pairs (Table 1). When we binned our dataset according to different dN ranges as mentioned previously, the trend remains the same for each dN range (Table 1, Fig. 2), which indicates that the SSD genes are more often colocalized, and WGD genes are significantly more diverged in their subcellular localization, irrespective of their sequence divergence.

### Gene expression correlation between SSD and WGD pairs

**Fig. 3:**
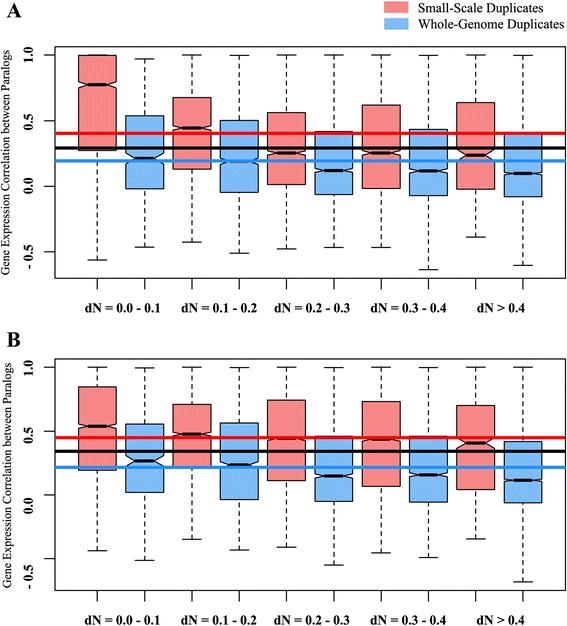
Differences in gene expression correlation between human small-scale and whole-genome duplicate pairs. The gene expression correlation values of SSD and WGD pairs were calculated using RNA-seq gene expression data from **a.** Human Protein Atlas and **b**. Expression Atlas. The SSDs are represented in brick red and WGDs are represented in blue. The red and blue lines represent the mean gene expression correlation of SSD and WGD pairs, respectively. The black line represents the mean of gene expression correlation of all human duplicated gene pairs. (For every dN range, *P* < 0.05). Exact P-values are provided in Table 1

The divergence of duplicated genes and can also occur at the gene expression levels. Earlier studies suggested that the gene expression patterns of duplicated pairs often undergo a spatial variation [reviewed in Li et al. [15]], and this can be considered as a mechanism for their stable maintenance [13]. Therefore, it is essential to understand the co-expression of the paralogs in different tissues after gene duplication, which is measured using the gene expression profiles of the paralogous copies in a wide range of normal tissues [14–16]. We used the high-throughput recent RNA-seq gene expression data of a wide range of normal human tissues from the Human Protein Atlas [49] and Expression Atlas [50] (see Materials and methods for more details). However, we observed that human SSD pairs have higher expression profile similarity than the WGD counterparts as a whole, and in each dN range (Table 1, Fig. 3), suggesting that the functionally redundant human SSD genes also have more correlated expression profiles, and WGDs tend to diverge more in gene expression patterns.

### Evolutionary rate of human SSD and WGD genes

**Fig. 4:**
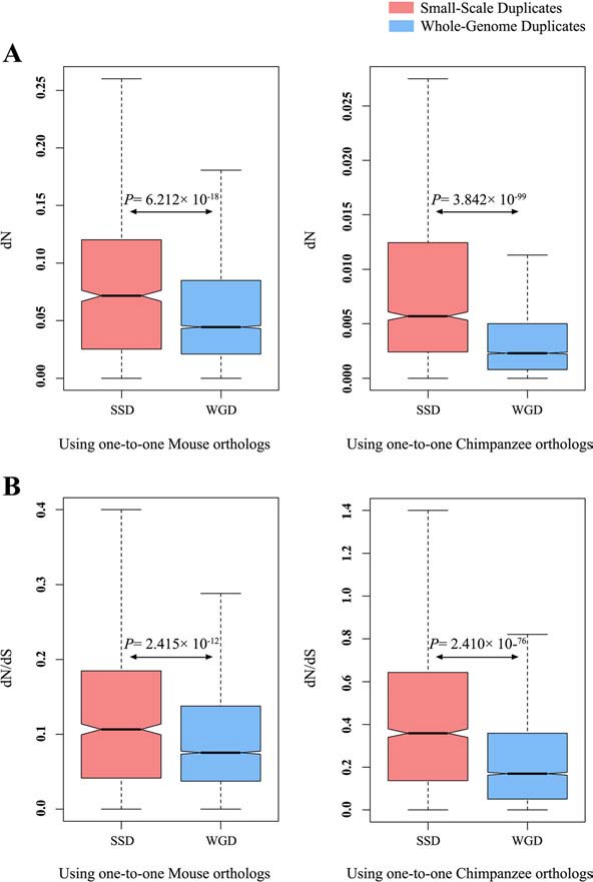
Differences in evolutionary rates of human small-scale and whole genome duplicates using Mouse (*Mus musculus*) and Chimpanzee (*Pan troglodytes*) one-to-one orthologs. Both the dN (**a**) and the $\frac{dN}{dS}$ ratios (**b**) were used as the measurements of evolutionary rate. The SSDs are represented in brick red and WGDs are represented in blue. Exact P-values are provided in the figure and in Table 2

The differences of human SSD and WGD pairs in their evolutionary genomic attributes clearly suggest that the human WGDs may tend to adapt themselves to new functions and locations. To investigate this, we used the one-to-one Mouse as well as Chimpanzee orthologs (see Materials and methods for details) to compare the evolutionary rates of human SSD and WGD genes by the Nonsynonymous nucleotide substitution per nonsynonymous sites (dN) and the $\frac{dN}{dS}$ ratio, where 'dS' denotes synonymous nucleotide substitution per synonymous sites. We obtained a significantly slower evolutionary rate in WGD genes than the SSD genes for both the cases (Table 2, Fig. 4), indicating that the human WGD genes are evolutionarily more conserved, besides being functionally more diverged than the SSD genes, which is also supported by a previous study [51] and is consistent with the idea of the slower evolutionary rate of duplicated genes following their adaptation to new circumstances as described in Jordan et al.[43].

**Table 2:** The evolutionary rate differences between human small-scale and whole-genome duplication using mouse (*Mus musculus*) and chimpanzee (*Pan troglodytes*) as outgroups. Two-tailed *Mann–Whitney U-Test* was used for comparisons between groups

Outgroup Used	Gene Group	Number of genes	Mean dN	P-value	Mean $\frac{dN}{dS}$	P-value
Mouse (*Mus musculus*)	Human Small-Scale Duplicates	958	0.089	6.212× 10^−18^	0.135	2.415× 10^−12^
	Human Whole-Genome Duplicates	5689	0.062		0.101	
Chimpanzee (*Pan troglodytes*)	Human Small-Scale Duplicates	1611	0.012	3.842× 10^−99^	0.480	2.410× 10^−76^
	Human Whole-Genome Duplicates	5309	0.006		0.257

### Multifunctionality of human SSD and WGD genes

**Fig. 5:**
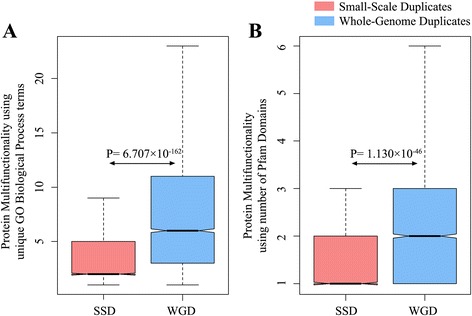
Multifunctionality of human small-scale and whole-genome duplicates: **a**. Using their association with unique GO-Biological Processes. **b**. Using the number of Pfam domains. The SSDs are represented in brick red and WGDs are represented in blue. Exact P-values are provided in the figure

The higher probability of functional, sub-cellular localization and gene expression divergence of human WGD genes and their evolutionary conservation suggests that they may be associated with miscellaneous functions in contrast to the SSD counterparts. As our study is based on the functional fates of SSD and WGD genes, we were interested to observe which group is associated with more numerous functions. We used the unique GO biological process terms [52, 53] and the Pfam domain count [54] as proxies of multifunctionality (see Materials and methods). We observed that WGD-only genes are associated with more numerous Gene Ontology terms [Mean number of unique GO terms in SSD ≈ 5, Mean number of unique GO terms in WGD ≈ 10, *P* = 6.707 × 10^−162^, *Mann Whitney U test*, N_SSD_ = 2569, N_WGD_ = 5437] [Fig. 5a] and contain significantly more domains in their encoded proteins [Mean number of Pfam domains in SSD = 1.61, Mean number of Pfam domains in WGD = 2.02, *P* = 1.130 × 10^−46^, *Mann Whitney U test*, N_SSD_ = 3060, N_WGD_ = 5607] [Fig. 5b] than SSD-only genes. This suggests that human whole-genome duplicates are associated with more variety of functions than human SSD genes.

### Gene essentiality between human SSD and WGD genes

So far, the comparison between the human SSD and the WGD genes showed that the SSD genes tend to diverge less in their function, subcellular localization, as well as in gene expression levels in different tissues. Additionally, WGD genes were also found to be evolutionarily more conserved and were adapted to new functions. But the importance of such functions from organismal perspective also plays a crucial role to get the whole picture. The importance of a gene can be measured in terms of gene essentiality. We used the Online GEne Essentiality (OGEE) Database [55] to obtain human essential genes [56] and observed a significantly higher proportion of essential genes among the WGD genes [Proportion of essential genes in SSD genes = 4.601 %, N_SSD_ = 2692; Proportion of essential genes in WGD genes = 11.344 %, N_WGD_ = 5730] [Z = −9.99, confidence level 99 %; *P* < 1.00 × 10^−4^, two sample Z-test]. In other words, a greater portion of WGD genes shows lethality or sterility upon deletion than SSD genes, due to the absence of redundant paralogs in the former group.

### Disease association of human SSD and WGD genes

Like gene essentiality, another important factor contributing to the importance of a gene in the organism is its disease association. It was previously hypothesised that gene duplication creates additional gene copies, and the increased functional redundancy can reduce the probability of disease formation by functional restoration upon gene deletion [57–59]. Therefore, the disease genes should remain as singletons [60]. More recently, studies linking gene duplication with disease hypothesise that duplication increase genetic redundancy, which in turn prefers accumulation of disease-associated mutations on the duplicates and hence, the duplicates may be more disease prone than the singletons [61]. Works with Mendelian disease genes reported their association with WGD genes [39, 62]. For our study, we considered all human disease associated genes from the Human Gene Mutation Database (HGMD) [63], which contains both Mendelian (monogenic) and complex (polygenic) disease genes. We observed that the proportion of disease genes is much higher among genes originating from whole-genome duplication [Proportion of disease genes in WGD genes = 61.46 %, N_WGD_ = 5908]; than the small-scale duplicates [Proportion of disease genes in SSD genes = 27.89 %, N_SSD_ = 3478] [Z = −31.420, confidence level 99 %; *P* < 1.00 × 10^−4^, two sample Z-test]. This suggests that the reduction of functional redundancy in WGD genes increases disease susceptibility, and the increased ability of functional restoration reduce disease association of SSD genes.

## Discussions

Gene duplication is the major source of genetic novelty that brings about genomic evolution. The term ‘genetic novelty’ comprise the generation of new genes from the pre-existing ones by mutation. Genetic mutation creates structural changes within the DNA which may lead to changes in the protein structure as well as its function. Although initially the duplicates are functionally redundant, they may either diverge or be maintained as backup copies during the course of evolution [2, 7, 64]. Recent studies with yeast confirmed that the whole-genome duplication maintains the stoichiometry of protein interaction network by increasing the dosage of its every participant, and small-scale duplication creates a stoichiometric imbalance within the network and hence, become functionally more divergent to maintain this balance [20, 38–41]. However, with the increasing organismal complexity and the genetic robustness, the whole-genome duplicates may also adapt to new functions, besides maintaining the resilience of protein interaction network. It will therefore be very interesting to explore the long-term fates of whole-genome duplication by observing human whole-genome duplicates (WGD), as the identifiable WGDs in human are traced from long back in the evolutionary scale i.e. from the two rounds of whole-genome duplication that had occurred in early vertebrate evolution . Therefore they must be evolved during the course of evolution from early vertebrates (like fish) to humans. In this study, we explored the distinguishable differences between human small-scale and whole-genome duplicates. As we mentioned, the small-scale (SSD) and whole genome duplicates (WGD) are not similar in terms of their origin, and therefore in sequence divergence. So, we binned our datasets according to the non-synonymous nucleotide substitutions (dN) to compare the similarities in evolutionary genomic properties between SSD and WGD duplicates independent of sequence changes that bring changes in amino acids, and in turn encoded proteins [20]. We observed that the human SSD and WGD duplicates were not similar in terms of their evolutionary and genomic properties. Based on their gene ontology terms, we found that WGD pairs share less functional similarity than the SSD pairs, irrespective of their sequence divergence for both the ‘GO Biological Process’ and ‘GO Molecular Function’ domains (Fig. 1, Table 1). We observed that these results are not influenced by duplicates having a large family size by conducting the same experiments using the closest duplicate pair only for both SSD and WGD duplicates (Additional file 1: Figure S1). We also observed that this difference is not due to the percentage identity based on which the SSD pairs are obtained, as using more stringent thresholds for determining SSD pairs also shows the similar trend (Additional file 1: Figure S2).

As the function of a protein is dependent on its localization in subcellular compartments [65], another possible mode of channelizing duplicated genes is in the subcellular localization of their encoded proteins [17]. Previous reports highlighted that the subcellular adaptation of duplicated proteins is also associated with the functional diversification [17, 47]. Consistent with this finding, we also observed a higher subcellular colocalization of the proteins encoded by SSD pairs (Table 1, Fig. 2; Additional file 1: Figure S3). This pattern is also opposite to that of yeast, where SSD pairs were more divergent in their subcellular localization, suggesting the human whole-genome duplicates have a higher probability of adapting themselves to new locations than the SSD counterparts. However, in higher eukaryotes having a tissue-level organization, gene duplication and the subsequent functional redundancy between the paralogs are often regulated by patterning their gene expression in different tissues [13–16, 66–68]. For example, the paralogs may express differentially in different tissues so that the amount of the produced protein remain at a steady level. Therefore, the spatial variation of gene expression can be treated as a possible candidate for the maintenance of duplicated pairs in humans. But the differences in gene expression patterns of SSD and WGD duplicated pairs were still not clear. Using high-throughput RNA-seq gene expression data of human for at least 27 normal tissues, we observed that the SSD pairs are more often coexpressed in the same tissue, whereas, WGD pairs tend to express differentially,  *i.e.* in different tissues. This explains the idea that these WGD duplicates have not only adapted themselves to divergent functions or new locations, but also in divergent tissues. This also suggests a higher probability of specialization of expression patterns of human WGD pairs than the SSDs having the same level of sequence divergence (Fig. 3, Table 1). Using more stringent sequence identity for identification of SSDs also shows the similar trend (Additional file 1: Figure S4). Additionally, using closest paralogs to normalize the influence of duplicates with large gene families also shows that the differences between human SSD and WGD pairs hold true (Additional file 1: Figure S1). However, as humans are very distantly related with reference to the vertebrate whole-genome duplication event, we hypothesised that our results reflect the long-term evolutionary fates of genes originating from whole-genome duplication, with those originating from small-scale duplications. To prove our hypothesis, we firstly explored the influence of recent small-scale duplications in our dataset uding phylostratum rank as the age of SSD genes [69]. We differentiated the SSD pairs in two groups- young-SSD pairs and old-SSD pairs (see Additional file 2 for more details) and reperformed our overall analysis. We observed that the proportion of young SSDs are very low in our dataset (Z = 79.875, confidence level 99 %; *P* < 1.00 × 10^−4^, two sample Z-test) and differences between old-SSD and WGD genes still persist (Additional file 2: Figure S5). From another perspective, we used *Xenopus tropicalis* as a control and compare the attributes of small-scale and whole-genome duplicates in xenopus genome. Interestingly, both the SSD and WGD pairs shows high functional similarity in xenopus, with very little or no difference (Additional file 3: Figure S6). This also indicates that in course of vertebrate evolution, although initially both the SSD and WGD duplicates were similar in their attributes, the WGD genes were found to be more suitable candidates to diverge themselves to perform novel functions.

The higher functional divergence of human WGD genes and divergence in their subcellular and tissue-specific gene expression patterns lead us to investigate the differences in evolutionary conservation between SSD and WGD genes. In general, the duplicated genes tend to evolve faster than singletons just after duplication due to the increased functional redundancy, and subsequently upon its functional specialization, these duplicates evolve at a slower rate to maintain the functions to which it became adopted [43]. However, the human WGD genes are identified as the genes originated at the early vertebrate lineage, where the two rounds of genome duplication had happened. We observed a slower evolutionary rate in human WGD genes in contrast to SSD counterparts, which clearly demonstrates that the WGD genes has become adapted to new functions and lost its redundancy, and became slow evolving to maintain these functions (Fig. 4). The slower evolutionary rates and higher functional divergence of WGD genes indicate that the functions, to which they are adopted, are also evolutionarily conserved.

Our hypothesis that human WGDs have adapted to divergent functions and became evolutionarily conserved is further strengthened by the analysis of protein multifunctionality. The WGD genes and their encoded proteins tend to have higher multifunctionality than the SSD genes (Fig. 5), which strengthen our idea of higher adaptation of human WGD genes to new functions in contrast to SSD counterparts. However, besides the functional fate of duplicated genes, we were interested to comprehend the importance of such functions to the organism’s life. Therefore, we also considered the importance of such functions to human. We used the gene essentiality along with the disease association as measurements of the vitality of a gene. Firstly we studied the effect of gene deletion to understand the functional restoration by the paralogous copy(ies). A recent study showed that the proportion of essential human gene is significantly higher in duplicates than in singletons [56]. Additionally, disease-associated genes were found to be enriched in duplicates [61]. Considering Mendelian disease genes, researchers also found WGDs to be more frequently disease-associated [39]. As our data contains two groups of duplicates which are quite different in their evolutionary genomic properties, we were curious to observe the proportion of essential genes and disease-associated genes (considering both Mendelian and Complex disease genes) among human SSD and WGD gene sets. We obtained a higher proportion of essential genes, as well as disease genes in the whole-genome duplicate set. Taken together, these results prove that the WGD genes have adapted themselves to serve more functions, which are more often crucial for humans, and may cause disease, sterility or even lethality upon disruption.

## Conclusions

In summary, our results suggest that the human duplicates originated from WGD event in early vertebrate evolution are quite different from those originating spontaneously at a smaller-scale (SSD), but these differences are exactly opposite to that of yeast. The possible explanation for this scenario is that the human WGDs have been traced back from long time ago on the evolutionary scale, as in humans, we obtained the WGDs from two rounds of whole-genome duplication occurred in early vertebrate lineage. Additionally, the SSDs in our dataset are also enriched in ancient genes. Clearly, it suggests that both the human SSD and WGD genes have faced many evolutionary challenges than that in yeast. However, we found that in long evolutionary timespan, WGDs are more prone to diverge themselves in structure, function as well as expression to perform new and beneficial roles within the organism than the SSD genes. This also increases the chance to cause disease or lethality upon mutation on the WGD genes, due to the inability of their paralogous copies to restore the gene-deletion fitness. However, why the ancient SSD and WGD genes show differences in their functional divergence, being evolutionarily similar in origin, is a matter of future investigation. In conclusion, our study provide an insight into the long-term evolutionary fate of duplicates originated from whole-genome duplication, rather than their immediate impact on the organism, to which the early studies with yeast [20, 41, 44] were focussed.

## Methods

### Identification of human small-scale and whole genome duplicates

We obtained 22,447 human protein coding genes from the biomart interface of Ensembl version 77 [46] (http://www.ensembl.org/biomart/martview). The whole-genome duplicate (WGD) pairs were obtained and compiled from two datasets: 1. Makino and McLysaght [39] and 2. OHNOLOGS (http://ohnologs.curie.fr/) [45]. We used the strict [q-score (outgroup) < 0.01 and q-score] (self comparison) < 0.01] dataset of OHNOLOGS database to discard false positives and maintain stringency of our data. All other duplicates were obtained from the biomart interface of Ensembl 77 [45] and termed as small-scale duplicates (SSD). We used 50 % sequence identity with high paralogy confidence to assign paralogs, in order to retain old and/or distant paralogs. Finally, we obtained 34,746 duplicated pairs with 21,446 SSD and 13,300 WGD pairs comprising 4670 and 7070 genes, respectively (Additional file 4: Table S1).

As our dataset contains two groups of duplicates originated differentially in evolutionary time-scale, they are also different in terms of sequence divergence between duplicated pairs. The whole genome duplicates have originated during the evolution of early vertebrates and the small-scale duplicates have originated spontaneously at different times, thus, the latter may contain more recent duplicates with a possibility of being less diverged in sequence level. Therefore, it is necessary to remove the bias due to the differential sequence divergence of SSDs and WGDs for calculating their differences in their functional properties. For this, we binned our dataset according to the nonsynonymous nucleotide substitution per nonsynonymous sites (dN) between each duplicated pairs, as the nonsynonymous substitutions bring change at protein level and older duplicate group (WGD) will tend to have higher dN than the newer one (SSD). Finally, both SSD and WGD duplicate pairs, we obtained five groups based on dN ranges between the paralogs − dN _0.0–0.1_, dN _0.1–0.2_, dN _0.2–0.3_, dN _0.3–0.4_ and dN _>0.4_ and differentiated the evolutionary features between SSD and WGD genes in each dN range.

### Functional similarity

The functions of human protein coding genes represented by their Gene Ontology terms were obtained from the biomart interface of Ensembl version 77 [46]. We considered the GO domains ‘Biological process’ as well as ‘Molecular function’ separately for functional similarity measurement. The functional similarity within each duplicate pair were calculated by their GO annotations using the following formula adapted from the Bayesian data integration method [44, 70]-

$$ \mathrm{Functional}\kern0.5em \mathrm{Similarity}\left(i,j\right)=\frac{\mathbf{2}\times \boldsymbol{S}\left(\boldsymbol{i},\boldsymbol{j}\right)}{\left[\mathbf{GO}\kern0.5em \mathbf{terms}\left(\boldsymbol{i}\right)+\mathbf{GO}\kern0.5em \mathbf{terms}\left(\boldsymbol{j}\right)\right]} $$

Where ‘i’ and ‘j’ are duplicated pairs and ‘S(i,j)’ represents the Gene Ontology terms shared between the duplicated pairs ‘i’ and ‘j’.

### Subcellular localization

The protein subcellular localization represented by the respective genes’ Gene Ontology terms for the GO domain ‘Cellular component’ were obtained from the biomart interface of Ensembl version 77 [46]. Considering the Gene Ontology terms of a gene and its paralog, we obtained the subcellular compartment sharing for each SSD and WGD duplicate pairs. With the same formula used for functional similarity calculation mentioned previously, we calculated the subcellular compartment sharing for each duplicate pairs and compared the SSD and WGD genes of different dN ranges (as mentioned above).

### Gene expression

The RNA-seq gene expression data of human were taken from two databases- The gene expression values of 9113 duplicated genes in 27 different tissues (namely adipose tissue, adrenal gland, appendix, bone marrow, cerebral cortex, colon, duodenum, oesophagus, gallbladder, heart muscle, kidney, liver, lung, lymph node, ovary, pancreas, placenta, prostate, salivary gland, skin, small intestine, spleen, stomach, testis, thyroid gland, urinary bladder and uterus) were extracted from the human protein atlas Release 9 (http://www.proteinatlas.org/) [49, 71] and 9393 duplicate genes in 32 different tissues (namely adipose tissue, adrenal gland, ovary, appendix, bladder, bone marrow, cerebral cortex, colon, duodenum, endometrium, oesophagus, fallopian tube, gall bladder, heart, kidney, liver, lung, lymph node, pancreas, placenta, prostate, rectum, salivary gland, skeletal muscle, skin, small intestine, smooth muscle, spleen, stomach, testis, thyroid and tonsil) were obtained from Expression Atlas (http://www.ebi.ac.uk/gxa) [50, 72], which present stable repositories of experimental RNA-seq gene expression data in human tissues. The Pearson correlation coefficient (see formula below) was used to determine the expression profile similarity within the paralogous copies.

$$ \mathrm{Pearson}\kern0.5em \mathrm{correlation}\kern0.5em \mathrm{coefficient}(r)=\frac{N{\displaystyle \sum ij}-\left({\displaystyle \sum i}\right)\left({\displaystyle \sum j}\right)}{\sqrt{\left[N{\displaystyle \sum {i}^2}-{\left({\displaystyle \sum i}\right)}^2\right]\left[N{\displaystyle \sum {j}^2}-{\left({\displaystyle \sum j}\right)}^2\right]}} $$

Where ‘*i*’ and ‘*j*’ are paralogous pairs, ‘N’ is the total number of tissues, ‘∑*ij*’ is the sum of the products of paired expression signal intensities, ‘∑*i*’ sum of expression signal intensities for gene ‘*i*’, ‘∑*j*’ is the sum of expression signal intensities for gene ‘*j*’, ‘(∑*i*^2^)’ is sum of squared expression signal intensities of gene ‘*i*’, ‘∑*j*^2^’ is sum of squared expression signal intensities of gene ‘*j*.

### Evolutionary rate

The oldest and widely used measurement of evolutionary rate calculates the evolutionary rate by using either dN values [73], or the $\frac{dN}{dS}$ ratio [74, 75], where 'dN' denotes Nonsynonymous nucleotide substitution per nonsynonymous sites and 'dS' stands for Synonymous nucleotide substitution per synonymous sites. For our study, we obtained one-to-one Mouse (*Mus musculus*) and Chimpanzee (*Pan troglodytes*) orthologs for each human genes to obtain the dN and dS values from the biomart interface of Ensembl version 77 [46]. Mutation saturation was controlled by discarding all dS values ≥ 3 [76]. We discarded the genes having paralogous copies from both small-scale and whole-genome duplications and used the nonredundant set of 9386 genes with only SSD or only WGD paralogs, but not both. Considering these SSD-only and WGD-only pairs, we obtained two distinct sets of genes: 1. Genes (and its paralogous copies) involved in Small-scale duplication only (SSD only) (containing 3478 genes), and 2. Genes involved in Whole-genome duplication only (WGD only) (containing 5908 genes). The dN values and $\frac{dN}{dS}$ ratios between these groups were compared and used as the measurement of evolutionary rate.

### Multifunctionality

The Multifunctionality of a gene and its encoded protein was measured by two approaches: A. Using their Gene Ontology annotation [77] for the GO domain ‘biological process’ from Ensembl Genome Browser [46], we calculated the unique biological processes of which a gene and its encoded protein(s) take part and used as the measurement of multifunctionality [51, 52], B. Additionally, we also considered the number of functional protein domains as proxy of Multifunctionality using Pfam protein families database. Finally, we compared the multifunctionality of SSD-only and WGD-only genes.

### Gene essentiality

The human gene essentiality data were obtained from the Online GEne Essentiality (OGEE) database (http://ogeedb.embl.de/#overview:) [55]. After matching this essentiality data with our dataset, we finally obtained gene essentiality information of 2692 SSD-only and 5730 WGD-only genes. We compared the proportion of essential genes between these duplicate sets.

### Disease association

Human disease genes were obtained from ‘The Human Gene Mutation Database’ (http://www.hgmd.cf.ac.uk/ac/index.php) [63]. After discarding redundancy, we were able to identify 9668 disease genes of which, 9299 genes were matched to our dataset. This contains both the monogenic and the polygenic disease genes and is considered as human disease-associated genes. All other genes were termed 'non-disease genes' (*N* = 13148). We compared the proportion of disease genes among the SSD-only (*N* = 3478) and WGD-only (*N* = 5908) sets.

### Software

We used the SPSS package (version 13) [78] and our in-house PERL-script for all statistical analyses. The R package [79] was used for data representation.

### Availability of supporting data

The dataset of human small-scale and whole-genome duplicate pairs used in the study is available in Additional Table S1.

### Ethics statement

The human data used in the study were collected from publicly available databases. Therefore ethics was not required for our study.

## Additional files

Additional file 1:
**Figure S1.** The differences between human small-scale and whole-genome duplicate pairs using the closest paralogs. **Figure S2.** Functional similarity between human small-scale duplicates with different sequence identity thresholds and whole-genome duplicate pairs. **Figure S3.** Subcellular co-localization between human small-scale and whole-genome duplicate pairs. **Figure S4.** Differences in gene expression correlation between human small-scale and whole-genome duplicate pairs. (PDF 662 kb) Additional file 2: Contains **Figure S5.** The differences between human young small-scale duplicates (Young-SSD) and old small-scale duplicates (Old-SSD) with whole-genome duplicates. (PDF 209 kb) Additional file 3: Contains **Figure S6.** The differences between *Xenopus tropicalis* small-scale and whole-genome duplicates. (PDF 379 kb) Additional file 4: Contains **Table S1.** The human Small-Scale and Whole-Genome duplicate pairs used in the study. (XLSX 705 kb)

## Abbreviations

PPI: protein-protein interaction network
SSD: small-scale duplicates
WGD: whole-genome duplicates
GO: Gene ontology
dN: Nonsynonymous nucleotide substitution per nonsynonymous sites
dS: Synonymous nucleotide substitution per synonymous sites
