# Supplementary material for: Global analysis of human duplicated genes reveals the relative importance of whole-genome duplicates originated in the early vertebrate evolution
Source: BMC Genomics. 2016 Jan 22;17:71. doi: 10.1186/s12864-016-2392-0 (PMC4724117; doi:10.1186/s12864-016-2392-0)
Supplement: Additional file 1: — Figure S1. The differences between human small-scale and whole-genome duplicate pairs using the closest paralogs. Figure S2. Functional similarity between human small-scale duplicates with different sequence identity thresholds and whole-genome duplicate pairs. Figure S3. Subcellular co-localization between human small-scale and whole-genome duplicate pairs. Figure S4. Differences in gene expression correlation between human small-scale and whole-genome duplicate pairs. (PDF 662 kb) [file 12864_2016_2392_MOESM1_ESM.pdf]

Global Analysis of Human Duplicated Genes Reveals the Relative Importance of Whole-Genome Duplicates Originated in the Early Vertebrate Evolution

Running title: The differences of human small-scale and whole-genome duplications

Authors:

Debarun Acharya[1] and Tapash C Ghosh[1]*

Affiliation:

[1]Bioinformatics Centre, Bose Institute, P 1/12, C.I.T. Scheme VII M, Kolkata 700054, West Bengal, India.

Tel.: +91-33-2355 6626; fax: +91-33-2355- 3886

*Corresponding author. To whom correspondence should be addressed.

E-mail address: tapash@jcbose.ac.in

Additional File 1:

Additional Figure 1

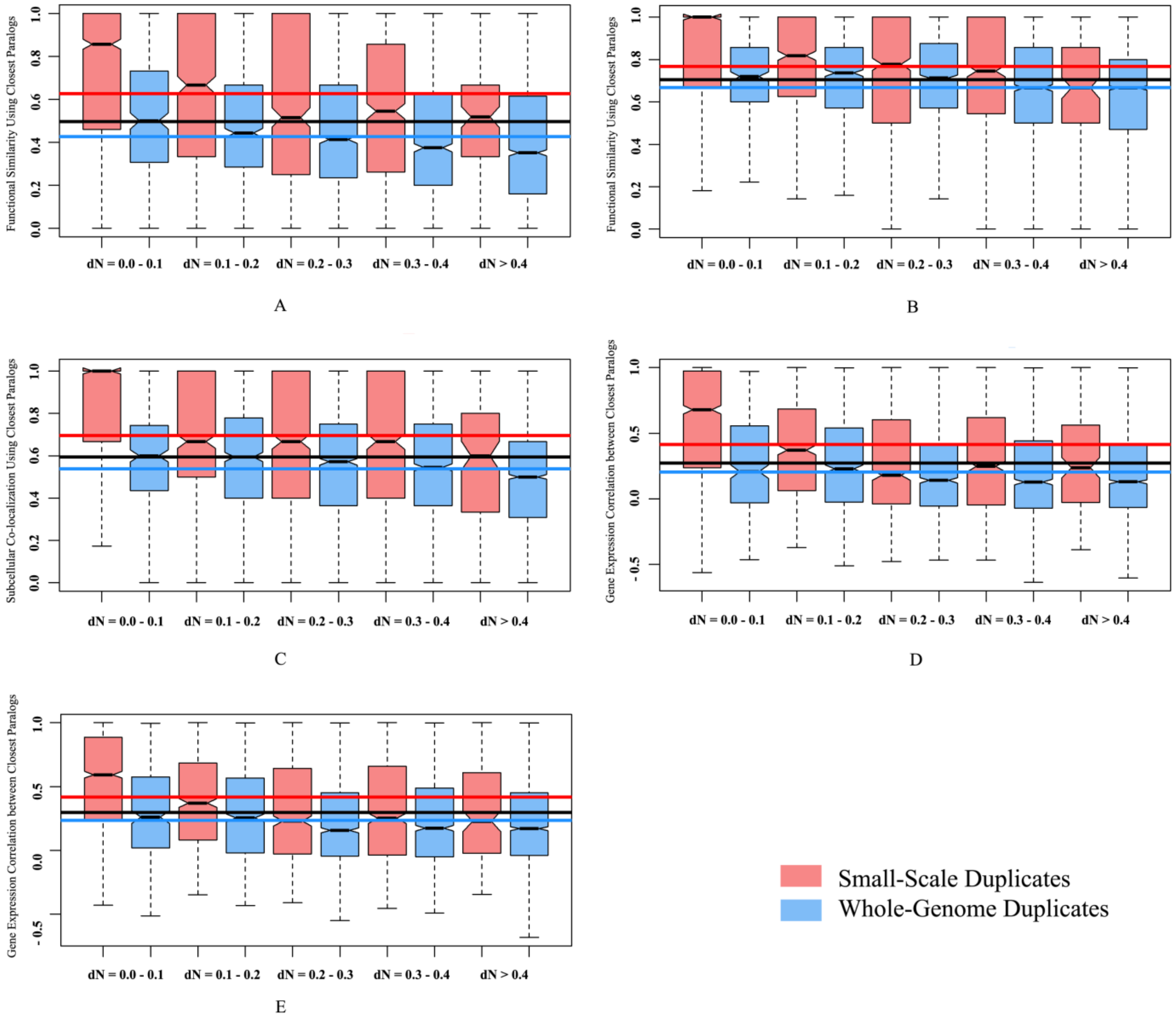

Additional Figure 1: The differences between human small-scale and whole-genome duplicate pairs using the closest paralogs. The SSDs are represented in brick red and WGDs are represented in blue. The red and blue lines represent the mean value of SSD and WGD pairs, respectively. The black line represents the mean value of all human duplicates. **A**. Functional similarity using GO domain 'Biological Process'; **B**. Functional similarity using GO domain 'Molecular Function'; **C**. Subcellular Co-localization using GO domain 'Cellular Component'; **D**. Gene expression correlation using 'The Human Protein Atlas'; **E**. Gene expression correlation using 'Expression Atlas'. The differences between SSD and WGD pairs in each bin is significant at least at 95% confidence limit (i.e., $P<0.05$).

Additional Figure 2

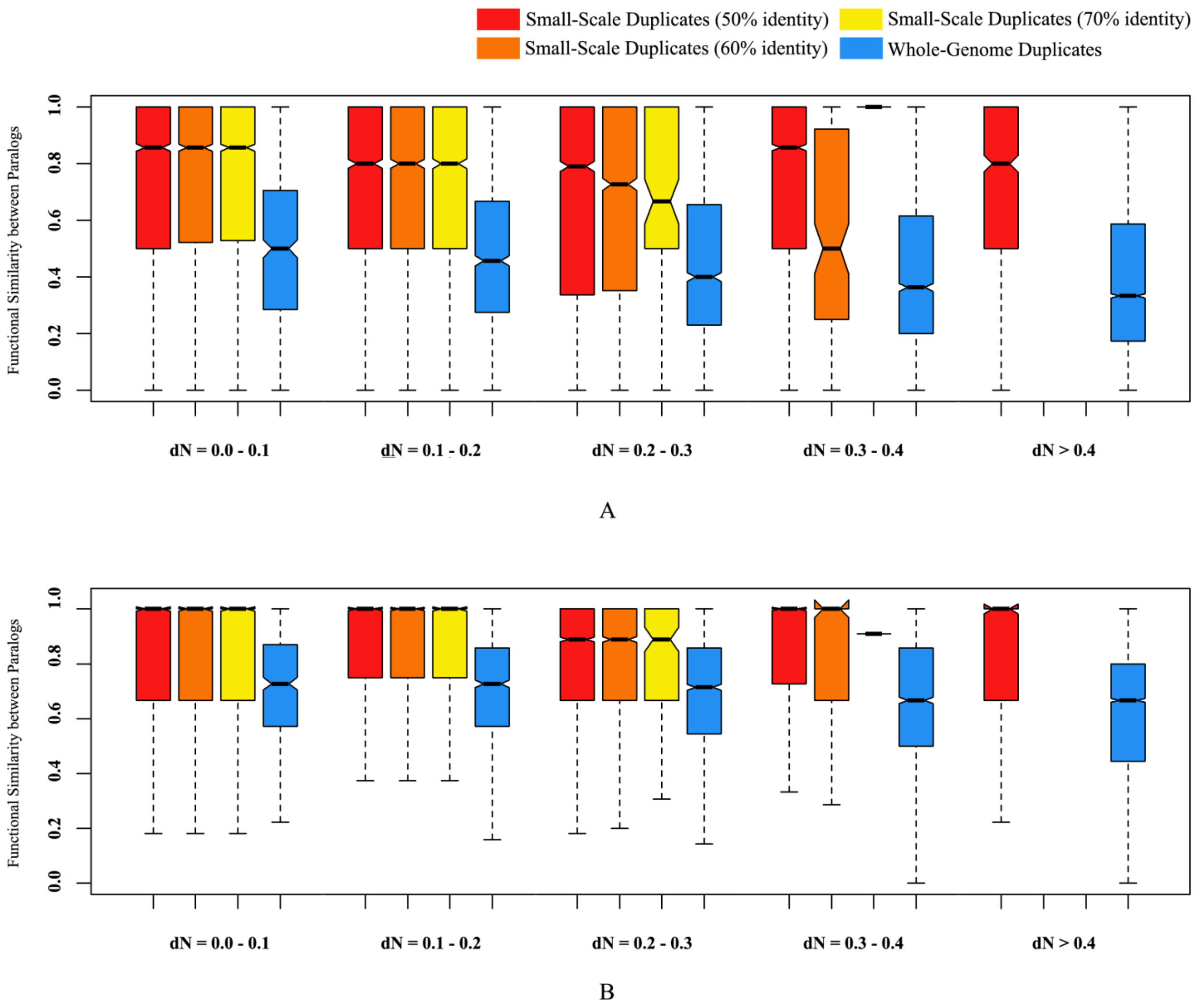

Additional Figure 2: Functional similarity between human small-scale duplicate pairs with different sequence identity thresholds and whole-genome duplicate pairs. Red, orange and yellow represents SSDs based on 50%, 60% and 70% sequence identity and blue represents WGDs. The functional similarities between different dN ranges were calculated using both GO domains **A.** Biological Process and **B.** Molecular Function. SSDs of each percentage identity group and WGDs in same dN range are different in at least 95% confidence limit (i.e. $P<0.05$).

Additional Figure 3

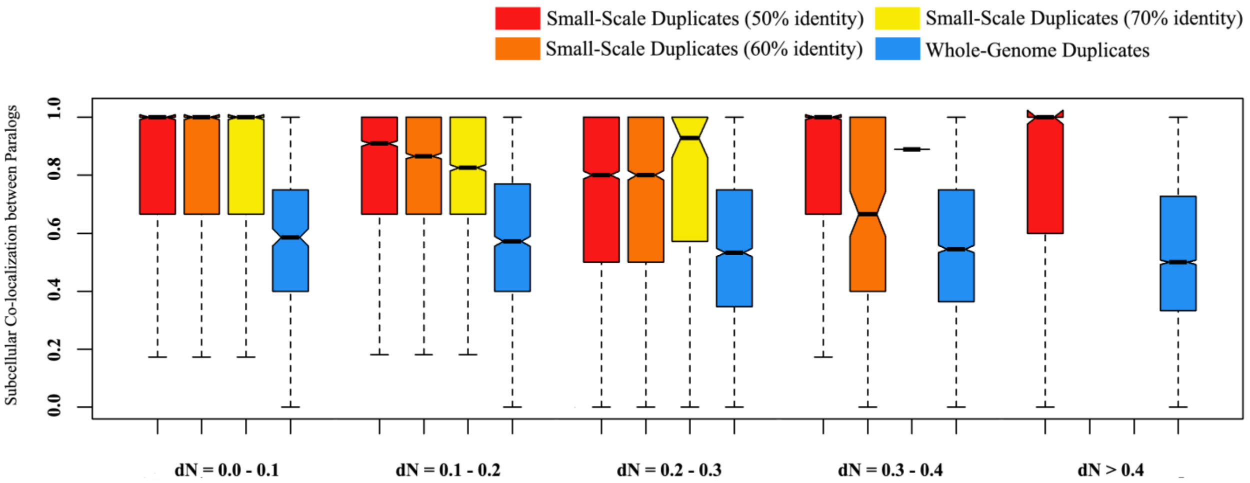

Additional Figure 3: Subcellular co-localization between human small-scale and whole-genome duplicate pairs. Red, orange and yellow represents SSDs based on 50%, 60% and 70% sequence identity and blue represents WGDs. SSDs of each percentage identity group and WGDs in same dN range are different in at least 95% confidence limit (i.e. P<0.05).

Additional Figure 4

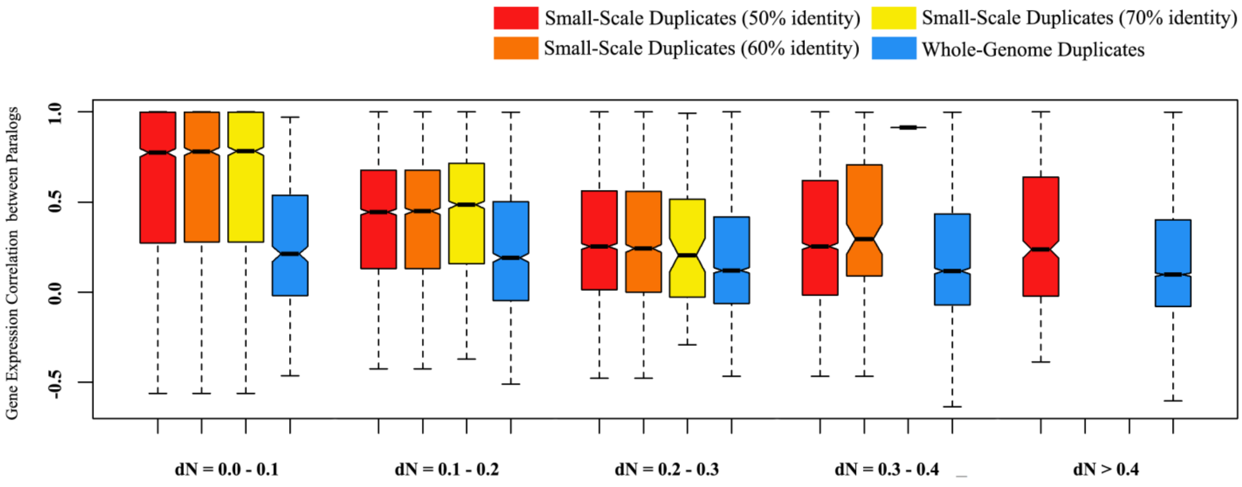

A

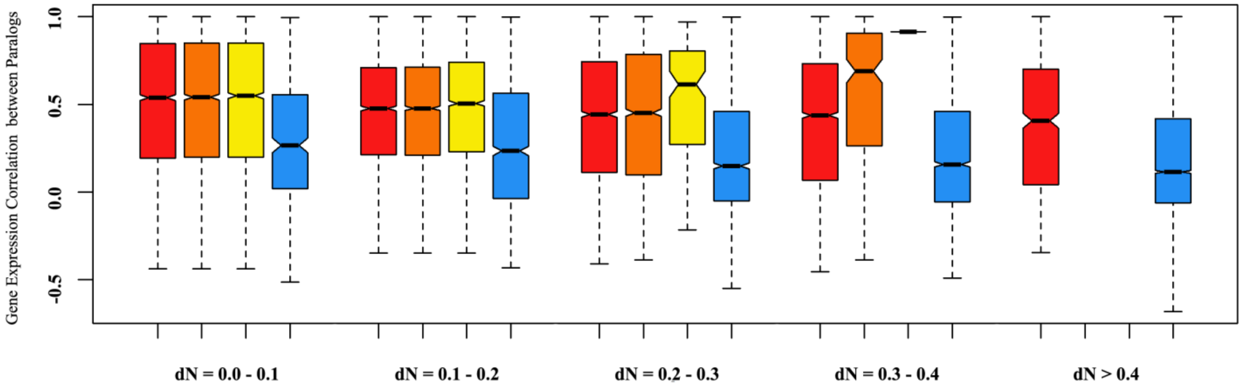

B

Additional Figure 4: Differences in gene expression correlation between human small-scale and whole-genome duplicate pairs. The gene expression correlation values of SSD and WGD pairs were calculated using RNA-seq gene expression data from **A.** Human Protein Atlas and **B.** Expression Atlas. Red, orange and yellow represents SSDs based on 50%, 60% and 70% sequence identity and blue represents WGDs. SSDs of each percentage identity group and WGDs in same dN range are different in at least 95% confidence limit (i.e. P<0.05).
