# Supplementary material for: Global analysis of human duplicated genes reveals the relative importance of whole-genome duplicates originated in the early vertebrate evolution
Source: BMC Genomics. 2016 Jan 22;17:71. doi: 10.1186/s12864-016-2392-0 (PMC4724117; doi:10.1186/s12864-016-2392-0)
Supplement: Additional file 2: — Contains Figure S5. The differences between human young small-scale duplicates (Young-SSD) and old small-scale duplicates (Old-SSD) with whole-genome duplicates. (PDF 209 kb) [file 12864_2016_2392_MOESM2_ESM.pdf]

Global Analysis of Human Duplicated Genes Reveals the Relative Importance of Whole-Genome Duplicates Originated in the Early Vertebrate Evolution

Running title: The differences of human small-scale and whole-genome duplications

Authors:

Debarun Acharya[1] and Tapash C Ghosh[1]*

Affiliation:

[1]Bioinformatics Centre, Bose Institute, P 1/12, C.I.T. Scheme VII M, Kolkata 700054, West Bengal, India.

Tel.: +91-33-2355 6626; fax: +91-33-2355- 3886

*Corresponding author. To whom correspondence should be addressed.

E-mail address: tapash@jcbose.ac.in

Additional File 2:

Comparison of human whole-genome duplicates with young and old small-scale duplicates:

Our study with human small-scale and whole-genome duplicates clearly suggests that these two groups of duplicates are quite different in their evolutionary genomic properties. To explain this, we hypothesized that our results reflect the long-term evolutionary fates of vertebrate whole-genome duplication. However, as the timing of duplication and subsequently the age of WGD and SSD duplicates may be different, we were interested to observe the proportion of recent and ancient duplicates among the SSD duplicates in our dataset. For this, we obtained the phylostratum gene age data from Neme and Tautz (2013)[1], where the human genes were ranked according to their earliest evolutionary origin. We classified all the SSD genes in our dataset into two groups- (A) Old SSD: representing all the genes before the emergence of eutherian mammals (having phylostratum rank 1-15) and (B) Young SSD: genes originated during eutherian lineage or later (having phylostratum rank 16-20). Mapping these two classes with our dataset of 4640 genes involved in small-scale duplication, we obtained 3888(95.29%) Old-SSD and 192(4.71%) Young-SSD genes. We mapped these Old- and New-SSD genes with our dataset of 21446 SSD pairs and obtained 14846 Old-SSD pairs and 642 Young-SSD pairs. We discarded the duplicated pairs where one gene is Old-SSD, and other is Young-SSD for maintaining stringency and facilitate pairwise comparison. We observed that both the Old- and Young-SSD genes show significant differences with WGD genes (**Additional Figure 5**). In addition to this, the low proportion of Young-SSD genes in our dataset clearly indicates that indeed our data of SSD pairs are not significantly enriched in younger genes (Z= 79.875, confidence level 99%; $P < 1.00 \times 10^{-4}$, two sample Z-test). Therefore, the differences between

human SSD and WGD duplicates really reflects the long-term fate of vertebrate whole-genome duplicates, in comparison with small-scale duplicates.

Additional Figure 5

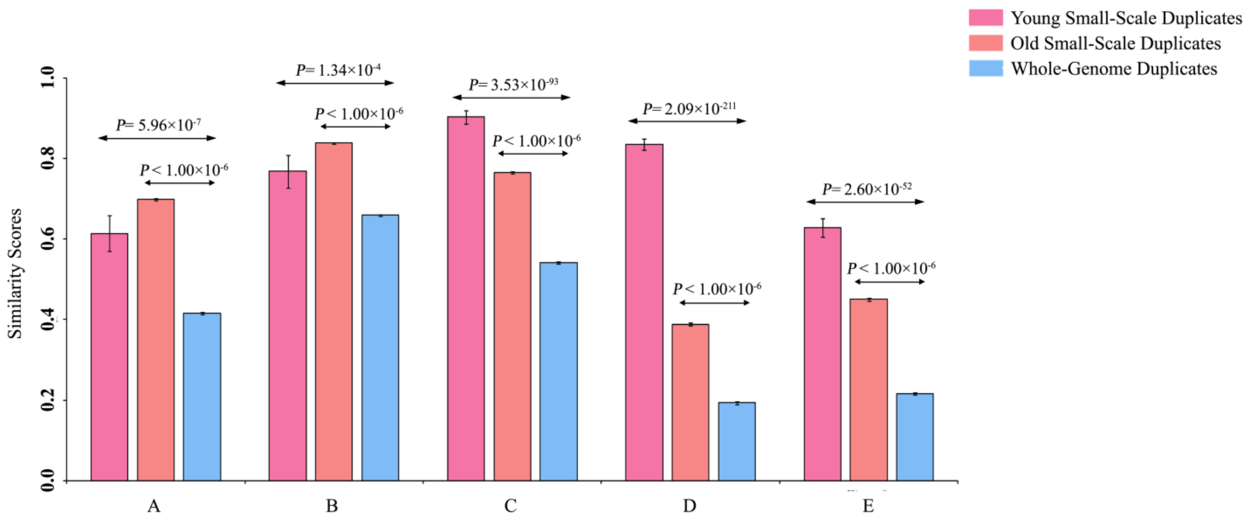

Additional Figure 5: The differences between human young small-scale duplicates (Young-SSD) and old small-scale duplicates (Old-SSD) with whole-genome duplicates (WGD). Y-axis represents the similarity scores of paralogous pairs. Young-SSDs, Old-SSDs and WGDs are represented in pink, brick red and blue, respectively. **A.** Functional similarity of duplicated pairs using 'GO Biological Process' annotation. **B.** Functional similarity of duplicated pairs using 'GO Molecular Function' annotation. **C.** Subcellular Co-localization of duplicated pairs using 'GO Cellular Component' annotation. **D.** Gene expression correlation among duplicate pairs using 'The Human Protein Atlas' data. **E.** Gene expression correlation among duplicate pairs using 'Expression Atlas' data. The P-values are provided in the figure.

References:

1. Neme R, Tautz D. **Phylogenetic patterns of emergence of new genes support a model of frequent de novo evolution**. BMC Genomics. 2013; 14(1):117.
