# Supplementary material for: Global analysis of human duplicated genes reveals the relative importance of whole-genome duplicates originated in the early vertebrate evolution
Source: BMC Genomics. 2016 Jan 22;17:71. doi: 10.1186/s12864-016-2392-0 (PMC4724117; doi:10.1186/s12864-016-2392-0)
Supplement: Additional file 3: — Contains Figure S6. The differences between Xenopus tropicalis small-scale and whole-genome duplicates. (PDF 379 kb) [file 12864_2016_2392_MOESM3_ESM.pdf]

Global Analysis of Human Duplicated Genes Reveals the Relative Importance of Whole-Genome Duplicates Originated in the Early Vertebrate Evolution

Running title: The differences of human small-scale and whole-genome duplications

Authors:

Debarun Acharya[1] and Tapash C Ghosh[1]*

Affiliation:

[1]Bioinformatics Centre, Bose Institute, P 1/12, C.I.T. Scheme VII M, Kolkata 700054, West Bengal, India.

Tel.: +91-33-2355 6626; fax: +91-33-2355- 3886

*Corresponding author. To whom correspondence should be addressed.

E-mail address: tapash@jcbose.ac.in

Additional File 3:

The difference between Small-scale and Whole-genome duplication in *Xenopus tropicalis* genome:

As human is very distantly related concerning whole genome duplication that had occurred in early vertebrate evolution, we compared WGD and SSD in *Xenopus* as a control to further strengthen our conclusion in human. In this study, we used our dataset of 34746 human duplicate pairs and mapped these with their one-to-one *Xenopus tropicalis* orthologs obtained from Ensembl biomart (version77) [1]. To retain the stringency of our data and avoid including false positive SSD duplicates in xenopus, we considered only the human orthologs for obtaining xenopus duplicate pairs. After this, we were able to obtain 1020 SSD and 8078 WGD pairs, respectively. Similar to our analysis in humans, the functional similarity between xenopus duplicated pairs were obtained using the Gene Ontology annotation for GO domains 'Biological Process' and 'Molecular Function'. The protein subcellular co-localization was measured using Gene Ontology annotation for GO domain 'Cellular Component'. We binned our dataset according to different dN ranges (as described in the Materials and Methods section in the manuscript) and compared SSD and WGD pairs within each dN range. We observed that, unlike humans, most of the differences are insignificant. We summarized all results in **Additional Figure 6**. For ease of understanding, the significant P-values were marked in red font.

Additional Figure 6

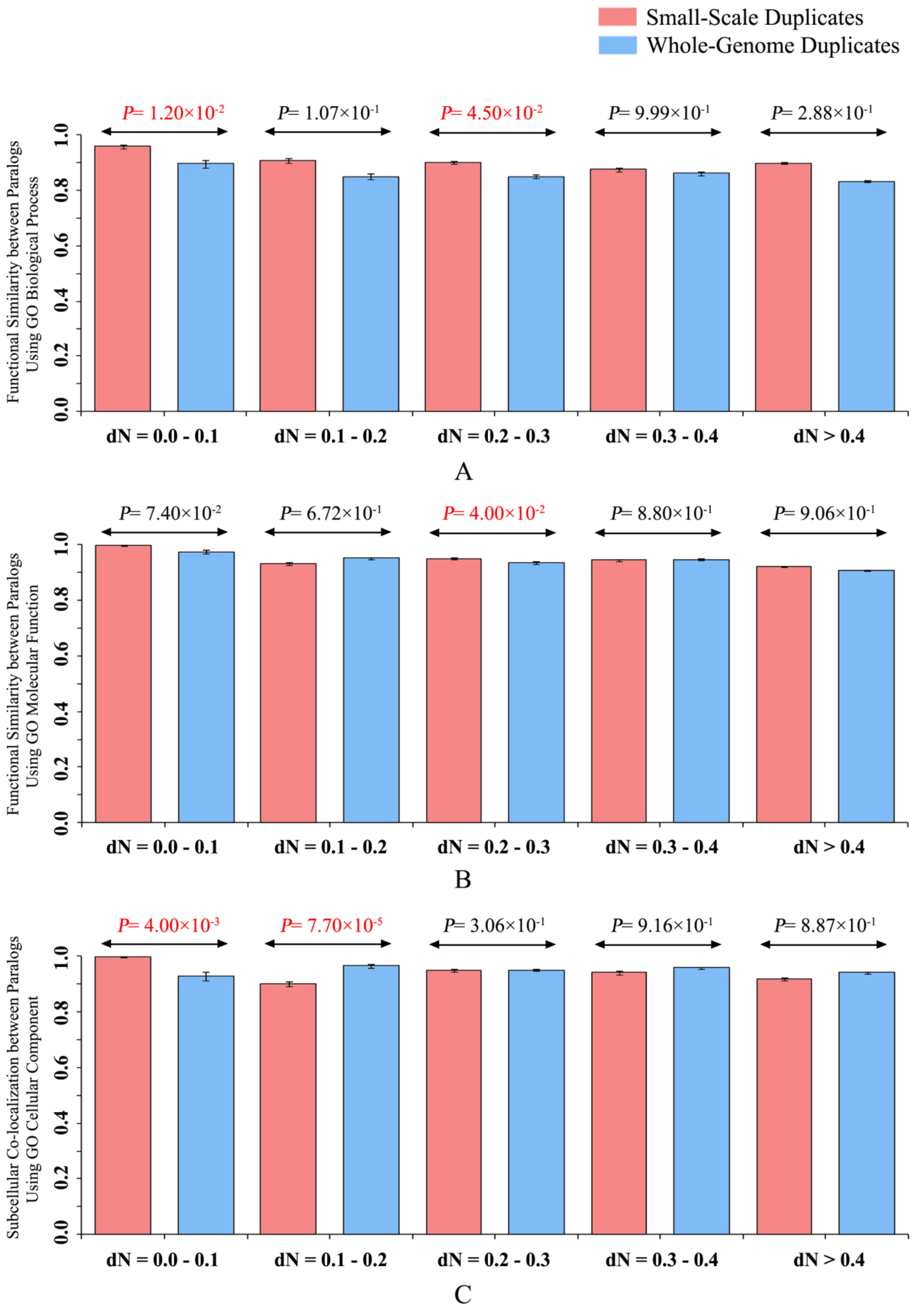

Additional Figure 6: The differences between *Xenopus tropicalis* small-scale (SSD) and whole-genome duplicates (WGD). SSDs are represented in brick red and WGDs are represented in blue. Significant P-values are marked in red. **A.** Functional similarity of xenopus SSD and WGD pairs using 'GO Biological Process' annotation. **B.** Functional similarity of xenopus SSD and WGD pairs using 'GO Molecular Function' annotation. **C.** Subcellular Co-localization of xenopus SSD and WGD pairs using 'GO Cellular Component' annotation.

References:

[1] Flicek P, Amode MR, Barrell D, Beal K, Billis K, Brent S, *et al*. **Ensembl 2014**. *Nucleic Acids Res.* 2014; **42**(D1):D749-D755.
